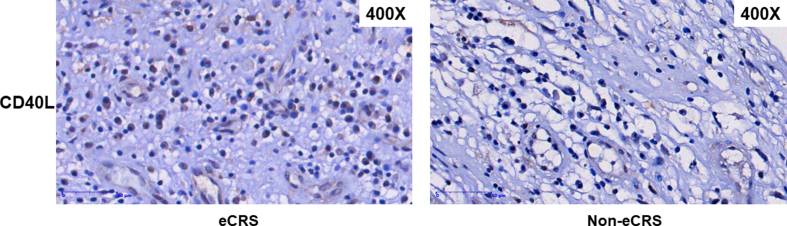# Assessment of serum soluble CD40 ligand levels in patients with chronic rhinosinusitis^☆^

**DOI:** 10.1016/j.waojou.2024.100880

**Published:** 2024-02-16

**Authors:** Zhichen Liu, Yuhui Fan, Aina Zhou, Jisheng Liu, Qingqing Jiao

**Affiliations:** a Department of Ear, Nose, and Throat, The First Affiliated Hospital of Soochow University, Suzhou, 215006, China; b Department of Dermatology, The First Affiliated Hospital of Soochow University, Suzhou, 215006, China

**Keywords:** Chronic rhinosinusitis (CRS), Eosinophilic chronic rhinosinusitis (eCRS), Soluble CD40-Ligand (sCD40L), CD40L, Inflammatory biomarker

## Abstract

Chronic rhinosinusitis (CRS) is a disease highly associated with abnormal regulation of T and B cells. The underlying pathophysiology of inflammatory pathways has critical implications for the diagnosis and management of CRS. Soluble CD40-ligand (sCD40L) is a cleaved form of CD40L present in plasma which functions the same way as CD40L, which has been observed as an inflammatory biomarker in many diseases. CD40L-positive cells control B-cell maturation, proliferation, apoptosis, and antibody production by binding to its receptor CD40 on B-cells. And our results show for the first time that patients with CRS have lower serum sCD40L levels compared to healthy subjects and that decreased sCD40L levels in patients correlate with increased CD40L-positive cell counts in the sinonasal mucosa. In addition, eosinophilic chronic rhinosinusitis (eCRS) patients tend to exhibit more CD40L-positive cells in the sinonasal mucosa compared to non-eCRS patients. This supports the notion that local blockade of CD40/CD40L may suppress pathogenic T/B cell responses and reduce tissue inflammation. Significantly, sCD40L and CD40L may be involved in the development and progression of CRS by impairing peripheral blood B-cell function and enhancing the local inflammatory response in the sinonasal mucosa.

Chronic rhinosinusitis (CRS) can be distinguished as eosinophilic (eCRS) and non-eosinophilic (non-eCRS) by the infiltration of eosinophils.^1^ The 2 subtypes differ in immunopathology and clinical management. eCRS is usually associated with more severe, recalcitrant disease and a poor prognosis.^1^ The underlying inflammatory pathways pathophysiology has necessary implications for the precise diagnosis and management of different subtypes of CRS.

B cells and their antibodies play a vital role in the pathogenesis of CRS. Inadequate B cell responses can increase susceptibility to CRS, while excess antibodies in local tissues can promote destructive inflammation.^2^ CD40/CD40 ligand (CD40L) is a co-stimulatory molecule of the tumor necrosis factor (TNF) superfamily. CD40L-positive cells control B-cell maturation, proliferation, apoptosis, and antibody production by binding to its receptor CD40 on B-cells.^3^^,^^4^ Soluble CD40-ligand (sCD40L) is a cleaved form of CD40L present in plasma which functions the same way as CD40L, which has been observed as an inflammatory biomarker in many diseases.^4^ In our previous study, we observed increased CD40L expression in nasal polyps of CRS patients.^5^ Still, it is intriguing whether sCD40L may be involved in the pathogenesis of CRS.

Therefore, in the present study, we have investigated whether serum sCD40L levels (i) differ in patients with different subtypes of CRS and healthy individuals and (ii) correlate with clinical parameters as well as the number of CD40L-positive cells in sinonasal mucosa innovatively.

Age- and sex-matched basically 30 CRS patients (including 14 eCRS and 16 Non-eCRS) and 20 healthy controls (HC) were enrolled in this study (Table 1). All CRS patients met the diagnostic criteria of the European Position Paper on Rhinosinusitis and Nasal Polyps 2020 (EPOS2020), and patients with eCRS and neCRS were grouped based on the EPOS2020 classification criteria. Patients with comorbidities of other infectious, inflammatory, and immunologic diseases not associated with CRS were excluded. HCs were not suffering from any infectious, inflammatory and immunologic diseases. All participants provided written informed consent in accordance with the Helsinki Declaration of the World Medical Association.

**Table 1:** Demographic and clinical profile of the subjects involving in the present study

	eCRS 14(12 M, 2F)	Non-eCRS 16 (11 M, 5F)	HC 20 (15 M, 5F)
Age (years, mean ± SD)	46.57 ± 16.21	42.69 ± 13.60	41.80 ± 12.91
sCD40L (ng/ml, mean ± SD)	10.60 ± 4.63	15.03 ± 4.74	17.10 ± 4.04
CD40L^+^cells/HPF (mean ± SD)	100.58 ± 47.85	39.05 ± 33.82	
Duration (months, mean ± SD)	45.43 ± 39.71	69.38 ± 146.41	
Lund-Kennedy score(mean ± SD)	2.90 ± 1.60	1.67 ± 2.25	
Eosinophil count (10^9^/L, mean ± SD)	0.25 ± 0.21	0.18 ± 0.25	
Eosinophil (%, mean ± SD)	3.46 ± 2.60	2.50 ± 3.62	
Eosinophil (cells/HPF, mean ± SD)	93.97 ± 74.56	4.12 ± 2.99	
CRSwNP, n (%)	13 (93%)	12 (75%)	
Comorbidity, n (%)			
Atopy	1 (7%)	1 (6%)	
Asthma	1 (7%)	1 (6%)	
Aspirin intolerance	0 (0)	1 (6%)	

Serum sCD40L levels were quantified using a commercial enzyme-linked immunosorbent assay (ELISA) kit (Bright Scistar, Suzhou, China). Sinonasal mucosa from the middle turbinate and uncinate process of CRS patients was collected under nasal endoscopy. However, sinonasal mucosa from healthy individuals could not be collected because removing normal sinus mucosa is not justified. Immunohistochemical stains of sinonasal mucosal CD40L was carried out. In addition, the Lund-Kennedy (LK) endoscopic score, Lund-Mackay (LM) score, and complete blood count results were collected.

Serum sCD40L levels were decreased in CRS patients compared to HC (12.96 ± 5.13 ng/ml vs 17.1 ± 4.04 ng/ml, p = 0.0026, Fig. 1A). Furthermore, the sCD40L levels were significantly lower in eCRS patients than in non-eCRS patients (10.6 ± 4.63 ng/ml vs. 15.03 ± 4.74 ng/ml, p = 0.015, Fig. 1B). In contrast, CD40L-positive cells were significantly higher in the sinonasal mucosa of eCRS patients than in patients of non-eCRS (100.58 ± 47.85 cells/HPF vs 39.52 ± 32.64 cells/HPF, p = 0.001, Fig. 1C and Supplementary Fig. 1). Our correlation analysis results show a modest negative correlation between serum sCD40L and sinonasal mucosa CD40L-positive cell level in CRS patients (r = −0.47, p = 0.017, Fig. 1D) and a more significant negative correlation between them in eCRS patients was further observed (r = −0.78, p = 0.011, Fig. 1E).

**Fig. 1:**
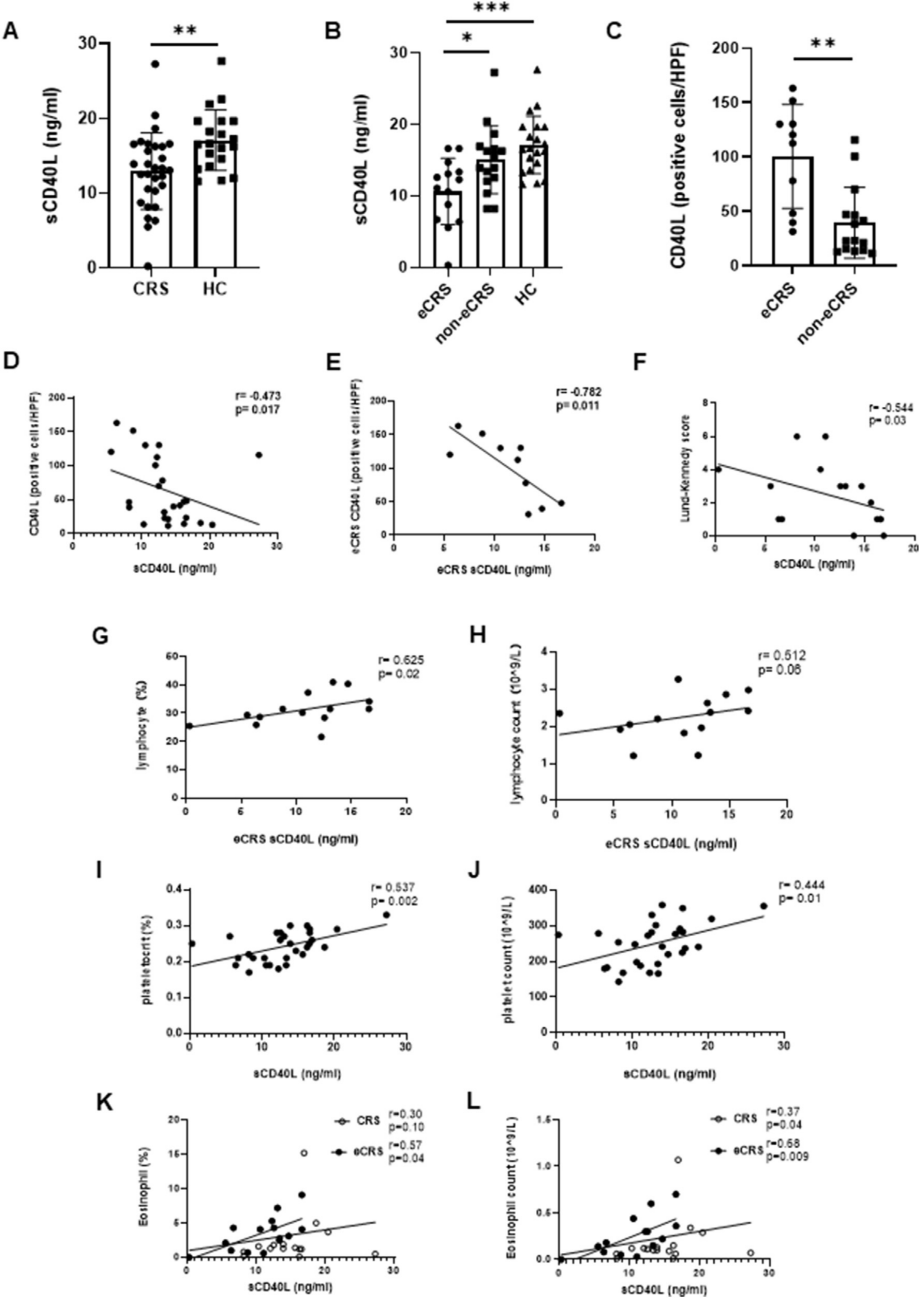
Expression levels of sCD40L in different subjects and correlation analysis of sCD40L levels with CD40L and clinical indicators in CRS patients

A significant negative correlation between sCD40L levels and Lund-Kennedy (LK) endoscopic score (reflecting the severity of nasal endoscopic inflammatory manifestations) was observed in CRS patients (r = −0.54, p = 0.03, Fig. 1F). In addition, a significant positive correlation between lymphocyte percentage and sCD40L levels was found only in patients with eCRS (r = 0.63, p = 0.02, Fig. 1G), whereas a possible correlation was found between lymphocyte counts and sCD40L levels (r = 0.51, p = 0.06, Fig. 1H). We further found that plateletocrit (r = 0.54, p = 0.002, Fig. 1I) and platelet count (r = 0.44, p = 0.01, Fig. 1J) were positively associated with sCD40L levels in all CRS patients. In addition, we showed that peripheral blood eosinophils in CRS patients appeared to be positively correlated with sCD40L levels (percentage: r = 0.30, p = 0.10; count: r = 0.37, p = 0.04; Fig. 1K-L), and this trend was significant in eCRS patients (percentage: r = 0.57, p = 0.04; count: r = 0.68, p = 0.009; Fig. 1K-L).

Both sCD40L and CD40L are involved in the CD40/CD40L axis, which plays a vital role in immune activation and regulating tissue inflammation.^3^ We observed that decreased serum sCD40L levels and increased sinonasal mucosa CD40L-positive cells in CRS patients. In addition, we found that lower sCD40L levels were associated with more severe endoscopic clinical manifestations in CRS patients. Also, lower serum sCD40L levels with higher levels of mucosal CD40L-positive cells were characteristic of eCRS compared to non-eCRS. Our data indicates that CD40L contributes to the immune response to CRS.

Different from previous findings that sCD40L levels are elevated in patients with several autoimmune diseases.^7^ CRS, as a non-autoimmune disease, in which the significance of the lower sCD40L expression levels is intriguing. The significant positive correlations between sCD40L levels and lymphocyte percentage, as well as plateletocrit and platelet count, may indicate that they are the primary source of serum sCD40L levels in CRS patients. In conjunction with previous studies, the lower sCD40L levels in CRS may be attributed to (i) an increased internalization of CD40L in the membranes of activated lymphocytes and (ii) a reduction in the release of sCD40L from platelets, eosinophils or activated T cells.^3^^,^^6^ Furthermore, the more significant positive correlation of lymphocytes and eosinophils with sCD40L in eCRS patients may suggest that lymphocytes and eosinophils have a more significant influence on sCD40L in eCRS than in non-eCRS. It may be attributable to the aberrant expression and activation of lymphocytes and eosinophils in the type 2 inflammatory response of eCRS, which is also a representative feature of eCRS.^1^

Both CD40L and sCD40L function by attaching to CD40 in B cells, which is essential for B cell proliferation, differentiation, antibody production, and costimulatory activity.^3^ Abnormal cell-mediated CD40L co-stimulation leads to aberrant B-cell activation, which induces pathogenic plasma cell, IgG/E/A production.^8^ Therefore, we hypothesize that reduced sCD40L levels may increase CRS susceptibility by impairing the activation of mature B cells in the peripheral blood of CRS patients. In turn, the increased abnormal activation of B cells mediated by CD40L-positive cells in local tissues may promote the development of destructive inflammation.^2^^,^^3^

Due to the limited number of subjects and originating from outpatients, there may be a risk of selection bias in this study. Still, we do not consider this to affect the overall trend of the results.

In summary, lower serum sCD40L levels and more sinonasal mucosa CD40L-positive cells are involved in the CRS disease process, probably by impairing peripheral blood B cells function and enhancing sinonasal mucosa inflammatory response, particularly in eCRS. Whether affecting CD40/CD40L pathway suppresses pathogenic immune responses and reduces tissue inflammation deserves to be explored.^1^ The role of sCD40L/CD40L in the pathogenesis of CRS and their clinical relevance should be evaluated in further studies.

## Abbreviations

CRS, Chronic rhinosinusitis; eCRS, eosinophilic chronic rhinosinusitis; Non-eCRS, non-eosinophilic chronic rhinosinusitis; CD40L, CD40 ligand; TNF, tumor necrosis factor; sCD40L, Soluble CD40-ligand; HC, healthy individuals; EPOS2020, European Position Paper on Rhinosinusitis and Nasal Polyps 2020; ELISA, enzyme-linked immunosorbent assay; LK, Lund-Kennedy; LM, Lund-Mackay.

## Funding

This study was partly funded by the National Science Foundation of China (Grants 82171159 and 82073434).

## Data statement

Raw data were generated at The First Affiliated Hospital of Soochow University, Suzhou, China. Derived data supporting the findings of this study are available from the corresponding author Liu J and Jiao Q on request.

## Authorship

Zhichen Liu has analysed the samples, contributed substantially to the conception and design and was involved in drafting the manuscript. Yuhui Fan has collected patient data and were involved in data analysis. Aina Zhou coordinated the histological pathology part of the study. Jisheng Liu was involved in proofreading the manuscript. Qingqing Jiao was the overall study coordinator and was involved in proofreading the manuscript.

## Ethics approval

Ethical approval from the Ethics Committee of The First Affiliated Hospital of Soochow University (Suzhou, China, No. 2022–215) was obtained prior to the study.

## Authors’ consent for publication

All authors have approved the final version of this manuscript and have been asked to give their consent to publication.

## Declaration of competing interest

The authors confirm that there are no conflicts of interest.

## Figures and Tables

**figs1:**